# Novel *APC* promoter 1B variant associated with gastric adenocarcinoma and proximal polyposis of the stomach: a case report

**DOI:** 10.3389/fgene.2026.1695311

**Published:** 2026-02-25

**Authors:** Jong Sun Park, Sun Gyo Lim, Jongmun Choi, Sae-Mi Lee, Chang Ahn Seol, Seon-Yong Jeong, Eunkuk Park, Young Bae Sohn

**Affiliations:** 1 Department of Gastroenterology, Ajou University Hospital, Ajou University School of Medicine, Suwon, Republic of Korea; 2 GC Genome, Yongin, Republic of Korea; 3 GC Labs, Yongin, Republic of Korea; 4 Department of Medical Genetics, Ajou University Hospital, Ajou University School of Medicine, Suwon, Republic of Korea

**Keywords:** regulatory region of *APC*, promoter 1B, c.-181dupC, novel variant, *in vitro* functional study

## Abstract

**Introduction:**

Gastric adenocarcinoma and proximal polyposis of the stomach (GAPPS) is a rare autosomal dominant familial gastric cancer syndrome. GAPPS is caused by pathogenic variant in the regulatory region of *APC*. This study describes the first Korean case of GAPPS associated with a novel likely pathogenic variant in *APC* promoter 1B region.

**Methods:**

A 35-years-old female patient who have gastric polyposis extending from the fundus to the body of the stomach was evaluated. Sanger sequencing was performed to detect mutations in *APC* promoter 1B region. A familial segregation study and *in vitro* luciferase activity assay were conducted to assess the pathogenicity of the novel variant.

**Results:**

A novel variant, c.-181dupC heterozygote, in *APC* promoter 1B region was detected. The same variant was found in her father, who underwent gastrectomy for gastric cancer. The *in vitro* functional study revealed a significant decrease in the expression of the *APC* promoter 1B variant in both HEK 293 TN and HeLa cells.

**Conclusion:**

This is the first reported case of GAPPS in Korea, associated with a novel likely pathogenic variant in APC promoter 1B, linked to a significant reduction in gene expression.

## Background

Gastric adenocarcinoma and proximal polyposis of the stomach (GAPPS) is a rare familial gastric cancer syndrome with an autosomal-dominant pattern of inheritance (Tanizawa et al., 2016). GAPPS is caused by mutations in the regulatory region of *APC*, whereas familial adenomatous polyposis is associated with intragenic mutation of *APC* (Rohlin et al., 2011; Li et al., 2016; Tacheci et al., 2021). Several mutational hotspots in *APC* promoter 1B, including c.-125delA, c.-191T>C, c.-192A>C, and c.-195A>C, have been identified in individuals with GAPPS (Li et al., 2016; McDuffie et al., 2016; Beer et al., 2017; Mitsui et al., 2018; Tacheci et al., 2021).

The onset age of GAPPS ranges widely from 10 to 45 years, with the youngest affected family members present with gastric polyposis and ages 23–56 years for gastric adenocarcinoma (Rudloff, 2018). The first reported cases in Asia were two cases in Japanese families (Yanaru-Fujisawa et al., 2012), while the initial report in Europe was a Czech white family across three generations (Repak et al., 2016). Sporadic fundic gland polyps (FGPs) tend to show non-neoplastic features and are related to more common use of proton pump inhibitors (PPIs), whereas FGPs associated with polyposis syndrome are likely to show dysplasia and progress to adenocarcinomas (Freeman, 2008). A 41-year-old woman diagnosed with GAPPS, who had multiple liver metastases and underwent systemic chemotherapy, and her two older brothers underwent prophylactic laparoscopic total gastrectomy with D1 lymph node dissection without complications, as first reported in Asia (Matsumoto et al., 2022).

It is primarily characterized by fundic gland polyposis restricted to the oxyntic mucosa of the gastric body and fundus, with no evidence of polyps in the colorectum or duodenum. The condition is characterized by numerous polyposis, >100 polyps carpeting the proximal stomach or >30 polyps in a first-degree relative of another case, confined to unusual locations, mainly the greater curvature of the body and fundus, while sparing the lesser curvature of the body and antrum. The predominant polyps are FGPs, with some progressing to dysplasia and others evolving into a significant risk for gastric adenocarcinoma. The exclusion criteria were as follows: (1) other heritable gastric polyposis syndrome and (2) a medical history of long-term use of PPIs (Rudloff, 2018).

The primary goal of GAPPS treatment is to prevent the progression of gastric cancer. Although exact guidelines and surveillance programs are absent, algorithms for family members have been proposed. If pathogenic variants are found in family members with GAPPS, and symptoms such as dyspepsia are present, gastroscopy should be initiated before the age of 15, with colonoscopy surveillance at the age of 18. Individuals of any age with dysplasia should undergo prompt gastrectomy. Individuals who have more than 100 FGPs require careful, case-by-case decisions regarding gastrectomy, considering the patient’s opinion and quality of life (Tacheci et al., 2021).

## Case presentation

**FIGURE 1:**
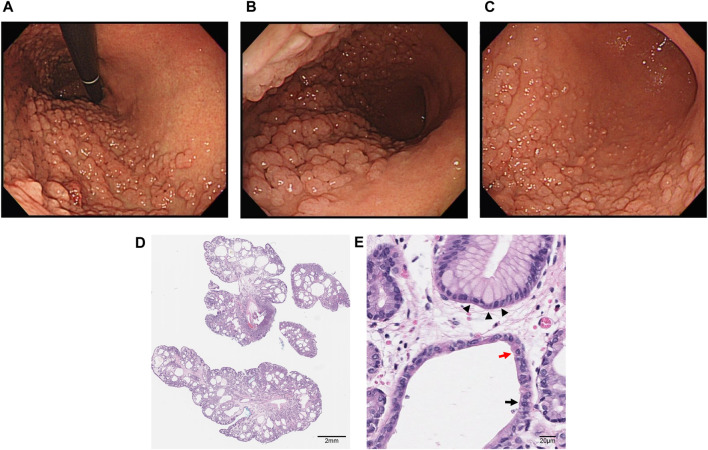
Endoscopic findings in the patient: numerous polyps of similar size located in the fundus **(A)** and greater curvatures as well as anterior and posterior walls of the gastric body **(B)**, sparing the gastric antrum and lesser curvature of the gastric body. High-definition gastroscopy with white-light imaging **(C)**. Histopathology of fundic gland polyps in GAPPS, stained with hematoxylin–eosin, original magnification ×20. Scale bar: 2 mm **(D)**. High-magnification histopathologic image of fundic gland polyps showing cystically dilated fundic glands lined by foveolar cells (black arrowheads), chief cells (black arrows), and parietal cells (red arrows). Scale bar: 20 μm **(E)**.

A 35 years-old female patient was referred to outpatient clinic of gastrointestinal department at Ajou University Hospital for gastric polyposis found during medical check-up. She had no known medical conditions and was not on any medication, including PPIs. The family history revealed, that her father had undergone gastrectomy for poorly differentiated gastric adenocarcinoma in the cardia at the age of 59. Endoscopic examination showed gastric polyposis consisting of >100 polyps extending from the fundus to the body, sparing the gastric antrum and the lesser curvature of the gastric body, with no evidence of duodenal involvement (Figures 1A–C). Histopathological analysis revealed cystically dilated glands lined by fundic-type epithelium composed of foveolar, chief, and parietal cells, without evidence of dysplasia, confirming FGPs (Figures 1D, E). Histopathological analysis revealed microcysts lined by fundic epithelium and did not show any dysplasia (Figure 1D). Small bowel series was performed, which revealed no detectable polyp in the small bowel. Colonoscopy showed no abnormal findings except for a single benign polyp. The physical examination at the first outpatient visit did not reveal any unusual findings, and laboratory tests were normal.

**FIGURE 2:**
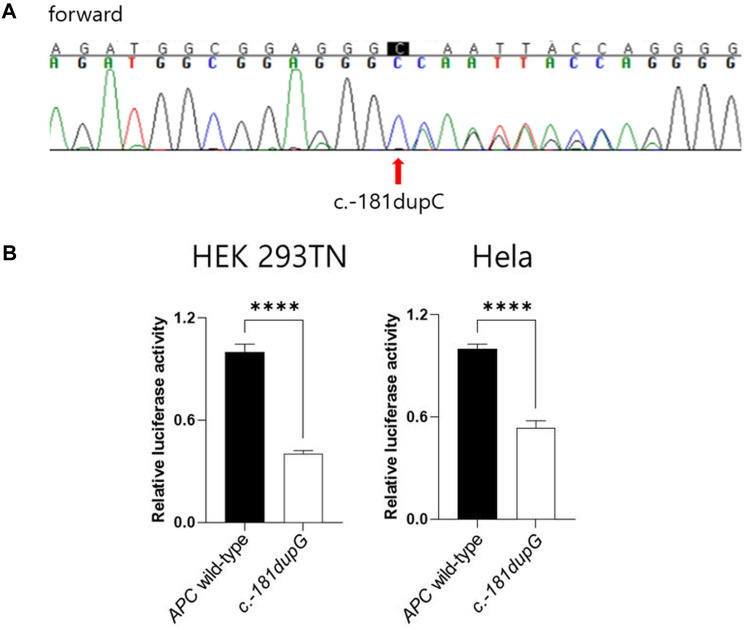
**(A)** Sanger sequencing results for *APC* promoter 1B showing heterozygous c.-181dupC variant. **(B)** Gene expression assay of the c.-181dupC variant in *APC* promoter 1B demonstrating significantly decreased expression in both HEK 293 TN and HeLa cells. *Firefly/Renilla* activity of the constructs was measured relative to that of the pRL *Renilla* vector. (****p < 0.00005, Student’s t-test).

Considering the development of gastric polyposis at a young age and the familial history of gastric cancer, next-generation sequencing (NGS) gene panel analysis for hereditary cancer, including *APC*, was initially performed, and found no pathogenic or likely pathogenic variants. As the NGS panel covered coding exons and exon-intron boundaries, Sanger sequencing of the *APC* promoter was subsequently performed. The sequence analysis identified a heterozygous c.-181dupC variant in *APC* promoter 1B, which was classified as a variant of unknown significance (VUS) (Figure 2A). To determine the significance of the VUS, further genetic familial segregation studies were conducted with the patient’s family (parents and younger brother). The same *APC* promoter variant was found in her father, who had a history of gastric cancer, whereas her asymptomatic mother and younger brother did not carry the VUS. Subsequently, an *in vitro* functional study was conducted to evaluate the pathogenicity of the VUS using a luciferase activity assay. Based on the sequence of the *APC* 1B transcript (GenBank accession No.: NM_001127511), the whole *APC* 1B promoter region (1042 bp) containing either the wild-type or variant sequence (c.-181dupC), was cloned into the pGL Basic vector (Promega). These constructs were co-transfected into HEK 293 TN and HeLa cells. Reporter gene expression was measured 24 h post-transfection using the Dual-Glo® Luciferase Assay System (Promega). The results demonstrated a significant decrease in the expression of *APC* promoter variant in both HEK 293 TN and HeLa cells (Figure 2B). Based on the *in vitro* assay results, the variant was reclassified as likely pathogenic. These results suggested that the GAPPS phenotypes in the patient were caused by the novel variant in *APC* promoter 1B region.

Prophylactic gastrectomy was recommended; however, the patient refused. The patient sought a second opinion at another tertiary care hospital regarding her gastric polyposis. She underwent upper gastrointestinal endoscopy, and biopsy results confirmed FGP without dysplasia or adenocarcinoma. Annual follow-ups were scheduled instead of prophylactic gastrectomy, and the patient did not present any new gastrointestinal symptoms or dysplasia in subsequent biopsy results.

## Discussion

We report the first case of GAPPS associated with a novel heterozygous likely pathogenic variant (c.-181dupC) in *APC* promoter 1B. The variant was co-segregated with the affected family member (her father). *In vitro* functional study revealed a significant reduction in mutant *APC* expression in both HEK 293 TN and HeLa cells compared to wildtype *APC*, supporting the pathogenicity of this novel variant. Previously reported pathogenic variants in *APC* promoter 1B, including c.-125delA, c.-191T>C, c.-192A>C, and c.-195A>C, reduce the binding of the Yin Yang1 (YY1) transcription factor and impair the activity of *APC* promoter 1B in the luciferase assay phenotype (Li et al., 2016). Furthermore, methylation studies have highlighted a selective gastric phenotype of *APC* promoter 1B variants compared to other familial adenomatous polyposis, mostly transforming to colorectal cancer, often associated with common exonic or splicing variants in *APC* (Li et al., 2016). Previously reported pathogenic variants in APC promoter 1B (e.g., c.-125delA, c.-191T>C, c.-192A>C, and c.-195A>C) have been shown to reduce YY1 binding and decrease promoter 1B activity (Li et al., 2016). However, the c.-181dupC variant identified in present study is outside the known core YY1 binding motif, and we did not directly assess YY1 binding for this specific variant. Therefore, although results of luciferase assays show that c.-181dupC significantly decreases promoter 1B activity, the exact mechanism is still unclear. Possible explanations include effects on binding of other transcription factors, changes of sequence or spacing that alter cooperative transcriptional control, and chromatin or epigenetic regulation such as methylation, associated with APC promoter 1B–related phenotypes (Rohlin et al., 2011; Li et al., 2016).

Clinically, the most important aspect of managing GAPPS is the early detection of dysplasia or adenocarcinoma and timely clinical intervention, rather than prevention of disease progression. This approach is essential for determining the optimal timing of prophylactic total gastrectomy, a procedure that has a substantial impact on long-term quality of life. In particular, for female patients of reproductive age with GAPPS, such as the 35-year-old patient in our case, decisions regarding the timing of prophylactic total gastrectomy require careful consideration, as postponement in favor of prolonged endoscopic surveillance may affect long-term prognosis and quality of life. However, the surveillance guidelines regarding the follow-up period for endoscopy and computed tomography or the optimal timing of prophylactic total gastrectomy, are not currently established. Furthermore, there is a case report of a 35-yearold woman who became pregnant and delivered a baby 2 years and 6 months after undergoing laparoscopic-assisted total gastrectomy. Two months after delivery, her serum AST and ALT levels began to rise. However, after transitioning from exclusive breastfeeding to a mixed feeding regimen of breast milk and formula, her liver function returned to normal levels. This case indicated careful monitoring and surveillance of iron and vitamin deficiencies together with liver function tests, during both the pregnancy and lactation in women who underwent total gastrectomy (Higashizono et al., 2018). In our case, the decision to postpone prophylactic total gastrectomy and the determination of the optimal timing for surgery significantly affect the patient's quality of life and clinical management, long-term prognosis, and potential complications associated with various types of gastric operations, including early and late dumping syndrome (Tanizawa et al., 2016). To summarize our findings, we identified a novel APC promoter 1B variant in a patient with GAPPS and demonstrated its functional impact by reduced promoter activity in luciferase assays, highlighting the importance of functional validation and individualized clinical decision-making. Given the presence of numerous polyps (>100) involving the body and fundus while sparing antrum in GAPPS, there is an increased risk of missing dysplasia and malignant transformation due to the technical difficulty in obtaining proper samples related to malignant changes. Furthermore, a case report describes acute progression of FGPs to gastric adenocarcinoma with systemic metastasis leading to death before prophylactic gastrectomy, and the two daughters from the same family underwent total gastrectomy with histopathology confirming adenocarcinoma at the ages of 23 and 30, respectively, which is notably younger than that in our case of a 35-year-old (Repak et al., 2016). Considering the case reports and the points discussed, early genetic counseling for first-degree relatives and family members with risk factors is essential to prevent delayed diagnosis. Early prophylactic gastrectomy should be considered on a case-by-case basis, along with clinical factors such as the type of gastrectomy, age, and sex (Tanizawa et al., 2016).

In conclusion, we report the first Korean case of GAPPS with a novel variant in *APC* promoter 1B. The pathogenicity of this variant was demonstrated by a luciferase activity assay in both HEK 293 TN and HeLa cells. Early identification of GAPPS through genetic consultation facilitates timely recognition of dysplasia or adenocarcinoma and guides appropriate clinical decision-making. Decisions regarding prophylactic total gastrectomy should therefore be individualized, taking into account multiple clinical factors on a case-by-case basis. To this end, multicenter collaborative studies involving multidisciplinary teams across advanced centers are needed to improve risk stratification and to establish evidence-based management strategies for GAPPS.

## Data Availability

The datasets presented in this article are not readily available because of ethical and privacy restrictions. Requests to access the datasets should be directed to the corresponding authors.
